# Immunomodulatory properties of *Bacillus subtilis* extracellular vesicles on rainbow trout intestinal cells and splenic leukocytes

**DOI:** 10.3389/fimmu.2024.1394501

**Published:** 2024-05-07

**Authors:** Samuel Vicente-Gil, Noelia Nuñez-Ortiz, Esther Morel, Cláudia R. Serra, Félix Docando, Patricia Díaz-Rosales, Carolina Tafalla

**Affiliations:** ^1^ Fish Immunology and Pathology Group, Animal Health Research Centre (CISA-INIA-CSIC), Madrid, Spain; ^2^ Centro Interdisciplinar de Investigação Marinha e Ambiental (CIIMAR), Universidade do Porto, Terminal de Cruzeiros do Porto de Leixões, Matosinhos, Portugal; ^3^ Departamento de Biologia, Faculdade de Ciências, Universidade do Porto, Porto, Portugal

**Keywords:** extracellular vesicles (EVs), *Bacillus subtilis*, probiotics, RTgutGC, cell line, B cells

## Abstract

Extracellular vesicles (EVs) are cell-derived membrane-surrounded vesicles that carry bioactive molecules. Among EVs, outer membrane vesicles (OMVs), specifically produced by Gram-negative bacteria, have been extensively characterized and their potential as vaccines, adjuvants or immunotherapeutic agents, broadly explored in mammals. Nonetheless, Gram-positive bacteria can also produce bilayered spherical structures from 20 to 400 nm involved in pathogenesis, antibiotic resistance, nutrient uptake and nucleic acid transfer. However, information regarding their immunomodulatory potential is very scarce, both in mammals and fish. In the current study, we have produced EVs from the Gram-positive probiotic *Bacillus subtilis* and evaluated their immunomodulatory capacities using a rainbow trout intestinal epithelial cell line (RTgutGC) and splenic leukocytes. *B. subtilis* EVs significantly up-regulated the transcription of several pro-inflammatory and antimicrobial genes in both RTgutGC cells and splenocytes, while also up-regulating many genes associated with B cell differentiation in the later. In concordance, *B. subtilis* EVs increased the number of IgM-secreting cells in splenocyte cultures, while at the same time increased the MHC II surface levels and antigen-processing capacities of splenic IgM^+^ B cells. Interestingly, some of these experiments were repeated comparing the effects of *B. subtilis* EVs to EVs obtained from another *Bacillus* species, *Bacillus megaterium*, identifying important differences. The data presented provides evidence of the immunomodulatory capacities of Gram-positive EVs, pointing to the potential of *B. subtilis* EVs as adjuvants or immunostimulants for aquaculture.

## 1 Introduction

Extracellular vesicles (EVs) are membrane-derived lipid bilayers which may contain cytosolic compounds as proteins and nucleic acids (1). The production of EVs has been reported in bacteria, but also in eukaryotic cells, including fungi and some parasites (2, 3). For years, EVs were considered metabolic or growth-related waste products (4) but nowadays have been demonstrated to have diverse biological functions. EVs are considered intercellular communication agents, able to affect host cell proliferation, gene expression and even cell death (5, 6). Among EVs, outer membrane vesicles (OMVs), which are specifically derived from Gram-negative bacteria, have been extensively characterized (7, 8). OMVs have been shown to contain specific membrane components, virulence factors, and DNA that encodes proteins involved in virulence, stress response, antibiotic resistance, and metabolism (9). Hence, OMVs aid in the promotion of pathogenesis, enable bacterial survival during stress conditions and regulate microbial interactions within bacterial communities (10). OMVs may also contain toxins, cytotoxic factors, lipopolysaccharides (LPS), and binding proteins that can signal host cells such as human cells (11–13). Consequently, OMVs have been sometimes seen to play a crucial role in infectious processes. Several reports have found that OMVs are involved in various inflammatory processes, as reviewed by Chen and colleagues (14). For example, it has been reported that *Helicobacter pylori* OMVs contribute significantly to inflammation by promoting the destruction of the mucin barrier (15). Similarly, *Moraxella catarrhalis* OMVs induce pulmonary inflammation in mice and can modulate the pro-inflammatory response of human epithelial cells (16).

Interestingly, OMVs not only provoke an inflammatory response but also affect the adaptive immune responses of a host. OMVs activate the immune system that recognizes in them pathogen-associated molecular patterns (PAMPs), ultimately affecting inflammation, immune gene transcription and cell proliferation (6, 17, 18). OMVs have also been shown to enhance phagocytic uptake (19). Taking advantage of their immunogenic properties, their use as adjuvants or even as vaccine platforms has been explored. Studies have shown that combining OMVs with proteins, LPS or even inactivated viruses can increase their immunogenicity and induce an efficient protective immune response. In one of these first studies, for example, meningitidis-derived OMVs complexed with *Shigella*-specific LPS were able to induce specific homologous anti-LPS antibodies in mice and provide immunity against *Shigella keratoconjuctivitis* in guinea pigs (20). Later studies have combined OMVs with inactivated respiratory syncytial virus (RSV) (21) or with malarial proteins (22) to develop intranasal vaccines. Remarkably, a vaccine against *Neisseria meningitidis* that is based on OMVs has already been licensed for human use by Novartis (known as Bexsero) (23).

For many years, it was believed that Gram-positive bacteria could not produce EVs due to the presence of a cell wall. However, nowadays, a wide variety of Gram-positive bacteria have been shown to produce EVs, including *Staphylococcus aureus* (24), *Listeria monocytogenes*, *Clostridium perfringens*, *Bacillus anthracis* (25, 26) and *Bacillus subtilis* (24). EVs of Gram-positive bacteria have been characterized by transmission electron microscopy and proteomic analyses. These Gram-positive EVs differed from those produced by Gram-negative bacteria in size. Hence, OMVs are generally larger in diameter (ranging from 20-200 nm) than Gram-positive EVs, which range from 20-100 nm in diameter (24, 27). Nonetheless, EVs share many properties of OMVs (24, 28). They have been also shown to be involved in cell communication and in sensing nutrients (29), and are known to significantly contribute to pathophysiological conditions in both bacteria–bacteria and bacteria–host interactions (27). Gram-positive EVs can also induce in the host both innate and adaptive immune responses, yet many aspects of the mechanisms by which Gram-positive EVs activate immunological responses in the host are still unclear (30). For example, both pro-inflammatory and anti-inflammatory effects have been reported for Gram-positive EVs (31–35).

Yet, some of the distinct features of Gram-positive EVs make them an even more appealing option to be employed as immunostimulants or potential vaccine platforms than OMVs. Gram-positive EVs are more stable in biological fluids and have shown differences concerning vesicle production and liberation (27). Unlike OMVs, Gram-positive EVs do not contain LPS, which may produce high toxicity in some species, thereby restricting their applications in highly sensitive species (28, 36). Thus, a number of studies in mammals have reported antibody production and increased survival when treated with EVs of pathogenic bacteria such as *S. aureus* (30, 37), *Streptococcus pneumoniae* (38), *B. anthracis* (31), *Enterococcus faecium* and *Enterococcus faecalis* (37), yet not many studies have addressed the immunostimulatory potential of non-pathogenic EVs (39).

In fish, only a limited number of works have explored the immune effects of EVs or their potential as vaccines, adjuvants or immunostimulants. Lagos and colleagues (40) demonstrated the presence of OMVs in the serum of *Piscirickettsia salmonis*-infected Atlantic salmon. Interestingly, a previous study, had shown that purified *P. salmonis* OMVs provoked a cytopathic effect on CHSE-214 (41). Similarly, OMVs from *Tenacibaculum dicentrarchi*, an important pathogen, were seen to be biologically active and induce a cytotoxic effect in macrophage-enriched cell cultures from rainbow trout head kidney (42). On the other hand, cyanobacteria-derived EVs were found to be harmless for zebrafish larvae, where they seemed to accumulate in the digestive tract (43). Then again, the proteome analysis of EVs derived from *Renibacterium salmoninarum*, a Gram-positive fish pathogen, showed a high abundance of major immunosuppressive proteins, such as P57/Msa and P22, as well as proteins associated with bacterial adhesion (44). The enrichment on P22 protein specifically suggested a role in host-pathogen interactions. Finally, *Flavobacterium psychrophilum* OMVs were capable of eliciting the transcription of immune genes related to the phagocytic, endocytic and antigen presentation pathways (45).

In this context, the focus of this study was to explore the immune effects of *B. subtilis* EVs on fish cells, with the general objective of searching for Gram-positive EVs with immunostimulatory capabilities that could eventually be used in the development of novel mucosal vaccines for aquaculture. We selected *B. subtilis*, an endospore-forming Gram-positive probiotic bacterium, commonly found in the gut of some aquacultured fish (46), given that the probiotic and immunostimulatory effects of *B. subtilis* in fish have been widely demonstrated, also in rainbow trout (47–49). For this, we have optimized a protocol for the physical separation of *B. subtilis* EVs and then evaluated the immunostimulatory properties of these EVs in a rainbow trout intestinal epithelial cell line (RTgutGC) and rainbow trout splenic leukocyte cultures. To our knowledge, this is the first work evaluating the immunomodulatory capacities of EVs produced by *B. subtilis* in rainbow trout cells, which reveal their potential as immunostimulants and adjuvants in aquaculture.

## 2 Material and methods

### 2.1 Isolation and characterization of *B. subtilis* EVs

The *B. subtilis* strain employed in this study was the ABP1 strain, previously isolated from European seabass (*Dicentrarchus labrax*) gut and defined as a probiotic (50). The ABP1 strain was grown in 500 ml of Luria-Bertani (LB) broth (Difco) at 25°C in an orbital shaker (100 rpm) for approximately 18 h, until the bacterial culture reached an exponential growth phase as determined by the optical density at 600 nm. EVs were then isolated by a physical separation protocol reported before (51), with minor modifications. Briefly, bacterial cells grown overnight were pelleted by centrifugation (10,000 x *g* for 20 min at 4°C). The supernatants were filtered through a 0.22 µm-pore-size filter (Millipore) and then concentrated by centrifugation on a Centricon Plus-70 filter Device (Millipore), followed by an additional filtration step. At this point, EVs were collected by centrifugation at 100,000 x *g* for 1 h at 4°C, being this the only step that was different to the previously described protocol (in which this centrifugation was performed at 150,000 x *g*). The pellets were resuspended in 75 μl of sterile and filtered phosphate-buffered saline (PBS) and stored at -80°C until use. In parallel, as a negative control, an LB medium without bacteria was subjected to the same process, to discard the possibility of lipids in the culture media forming vesicles. The sterility of all samples was assessed on LB plates.

In addition, the immunomodulatory properties of *B. subtilis* EVs were analyzed and compared with another strain of *Bacillus* species, namely *B. megaterium* (52). This species was grown in the same conditions and EVs were isolated in parallel, following the methodology just described.

In all cases, isolated EVs were characterized using different techniques. First, the protein concentration in the obtained preparations was determined using the Micro BCA™ Protein Assay Kit (Thermo Scientific). Thereafter, 18 μg of protein were mixed with 2X loading buffer [0.125 M Tris-HCl, 10% glycerol, 10% 2-mercaptoethanol, 100 mM dithiothreitol (DTT), 4% sodium dodecyl sulfate (SDS) and 0.05% bromophenol blue], boiled for 5 min and loaded on a 15% acrylamide gel containing 6 M of urea. After electrophoresis, the gel was stained with Coomassie blue. The size distribution and concentration of the EVs were analyzed by Dynamic Light Scattering (DLS) using the Zetasizer Ultra (Malvern analytical) device. DLS analysis allows quantitative and high-throughput characterization of vesicles in a low sample volume (53). The Zetasizer Ultra is equipped with a 633 nm He–Ne laser, operates at an angle of 173° and follows a mixed-mode measurement phase analysis light scattering (M3-PALS). For this, EVs were diluted 1,000-fold in PBS and loaded in a polystyrene cuvette (SARSTEDT). For each sample, three measurements were performed with standard settings (Liposomes; fixed position with an automatic attenuator; temperature of 25°C) and eventually averaged. The Zetasizer software reported intensity, size and concentration (particles/ml).

Finally, in the case of *B. subtilis*, the morphology of the isolated EVs was observed by transmission electron microscopy. For this, EVs were negatively stained with 2% uranyl acetate before visualization in a JEOL JEM 1400 Flash Transmission Electron Microscope (TEM) (Japan) operating at 100 kV. Images were recorded with an OneView CMOS digital camera (Gatan, USA).

### 2.2 *In vitro* stimulation of RTgutGC cell line

The intestinal epithelial cell line RTgutGC (Rainbow Trout gut Guelph Canada) has been isolated from the distal portion of the intestine of a rainbow trout (*Oncorhynchus mykiss)* (54). The cell line was cultured in Leibovitz’s medium (L-15, Gibco) supplemented with 100 I.U./ml penicillin and 100 μg/ml streptomycin (1% P/S, Life Technologies) and 10% fetal calf serum (FCS, Gibco) in T75 flasks at 19°C. The day before the stimulation, confluent cells were washed with medium, detached using trypsin (Gibco) and cell viability was determined using trypan blue (Sigma). Cells were then adjusted to 4 x 10^6^ cell/ml in complete L-15 medium (supplemented with antibiotics and 10% FCS). Subsequently, cells were seeded into 24-well plates (1 ml per well) and incubated for 24 h at 19°C. At this point, cells were stimulated with *B. subtilis* EVs to reach final concentrations of 3 x 10^5^, 1.5 x 10^6^ and 3 x 10^6^ particles/ml in the wells. As negative controls, cells were exposed to the same volume of the control preparation (LB subjected to the same process of EV isolation). Six replicate wells were included in all cases. After stimulation, cells were incubated at 19°C for 24, 48 and 72 h. At these points, RNA was extracted from the cells to determine the transcriptional response.

In some experiments, the effects of *B. megaterium* EVs on RT-gutGC cells were compared to those of *B. subtilis* EVs. For this, RTgutGC cells disposed in 24-well plates as described above were stimulated with 1.5 x 10^6^ particles/ml. Negative control cells were exposed to the same volume of the control preparation. In all cases, five replicate wells were included. In this case, cells were incubated at 19°C for 48 h before RNA extraction.

### 2.3 Experimental fish

Rainbow trout (*Oncorhynchus mykiss*) of approximately 400 g were obtained from *Piscifactoria Cifuentes* (Cifuentes, Guadalajara, Spain). Fish were maintained at the animal facilities of the Animal Health Research Centre (CISA-INIA-CSIC) in a re-circulating water system at 14°C with a photoperiod of 12:12 h light/dark and were fed twice a day with a commercial diet (Skretting Spain S.A). Animals were acclimatized for two weeks before they were used for experimentation. During this time, they were regularly checked for clinical signs, which were never observed.

### 2.4 *In vitro* stimulation of rainbow trout splenic leukocytes

Rainbow trout were sacrificed by benzocaine (Sigma Aldrich) overdose (50 mg/l) and peripheral blood was extracted from the caudal vein. The spleen was then collected and used to isolate total leukocyte populations. Hence, cell suspensions were obtained by passing the spleen through a 100 µm nylon mesh (BD Biosciences) using L-15 containing 1% P/S, 10 U/ml heparin (Sigma- Aldrich) and 2% FCS. Cell suspensions were then placed onto 30/51% Percoll (GE Healthcare) density gradients and centrifuged at 500 x *g* for 30 min at 4°C, without brake. The interface cells were collected, washed with L-15 supplemented with antibiotics and 2% FCS and resuspended in L-15 medium containing P/S and 5% FCS. The viable cell concentration was then determined by Trypan blue (Sigma-Aldrich) exclusion and cells were adjusted to a concentration of 2 x 10^6^ cells/ml. Depending on the experiment, splenic leukocytes were seeded in 24-well plates (Nunc) at a concentration of 2 x 10^6^ cells per well (for transcriptional studies) or in 96-well plates at 5 x 10^4^ cells per well (to study effects on IgM^+^ B cells). In all cases, splenocytes were exposed to EVs to make final concentrations of 3 x 10^5^, 1.5 x 10^6^, and 3 x 10^6^ particles/ml in the wells. Negative controls in which cells were exposed to the same volume of the control preparation were always included (LB subjected to the same process of EV isolation). Splenocytes were then incubated at 19°C for 24 h (for transcriptional studies) or 72 h (to study effects on IgM^+^ B cells).

### 2.5 RNA extraction, cDNA synthesis and gene expression analysis

RTgutGC cells and splenic leukocytes exposed or not to EVs were collected in 1 ml of TRI Reagent solution (Invitrogen), after discarding the culture supernatants. Total RNA isolation was performed following the manufacturer´s specifications. RNA quantification was performed with a NanoDrop 1000 Spectrophotometer (Thermo Fisher Scientific). One μg of RNA was then treated with DNase I (Invitrogen) and used to synthesize cDNA using the RevertAid Reverse Transcriptase (Thermo Fisher Scientific) with oligo (dT)_23_VN, following the manufacturer’s instructions.

Real-time PCR analyses were performed with the LightCycler96 System instrument (Roche) using FastStart Essential DNA Green Master reagents (Roche) and specific primers previously described (**Supplementary Table S1**). Each sample was incubated for 10 min at 95°C, followed by 40 amplification cycles (10 s at 95°C, 10 s at 60°C and 10 s at 72°C). A dissociation curve was obtained by reading fluorescence every degree between 60°C and 95°C to ensure only a single product had been amplified. The relative expression levels of the genes were normalized to the expression of *b-actin*, as a reference control gene. This reference gene was selected among two candidate genes after verifying that no statistical differences were detected among *b-actin* Ct values from different samples, following the MIQE guidelines (55). Expression levels were calculated using the 2^-ΔCt^ method, where ΔCt is determined by subtracting the *b-actin* value from the target cycle threshold. Negative controls with no template and *minus* reverse transcriptase controls were included in all cases.

### 2.6 Evaluation of the effect of *B. subtilis* EVs on IgM^+^ B cell populations by flow cytometry

We first evaluated how *B. subtilis* EVs affected the presence of different B cells in splenocyte cultures by flow cytometry. For this, splenic leukocytes incubated for 72 h with different doses of EVs or left unstimulated in the same conditions were harvested, washed with staining buffer (phenol red-free L-15 medium supplemented with 2% FCS) and then incubated for 1 h at 4°C with specific monoclonal antibodies (mAbs), anti-trout IgM (1.14) [mAb mouse IgG1 coupled to R-phycoerythrin (R-PE), 1 μg/ml] (56) and anti-trout IgD [mAb mouse IgG1 coupled to allophycocyanin (APC), 5 μg/ml] (57) diluted in staining buffer. Negative controls in which cells were exposed to the same volume of the control preparation were also included (LB subjected to the same process of EV isolation). After the incubation, cells were washed twice with staining buffer and counterstained with 0.2 μg/ml 4′, 6-diamidino-2-phenylindole (DAPI, Sigma) to determine cell viability. Only live cells were included in the analysis, which was performed on a FACS Celesta^™^ flow cytometer (BD Biosciences) equipped with FACSDiva software. In all cases, cells incubated with R-PE or APC-conjugated mouse IgG1 isotypes (clone MOPC-21, Biolegend) were used as controls, to confirm the specificity of the mAbs used. Doublets and dead cells were excluded from the flow cytometry analysis following the gating strategy described in **Supplementary Figure S1**. Data analysis was performed with FlowJo^®^ v.10 (Tree Star).

B cells are professional antigen-presenting cells that express major histocompatibility complex (MHC) II, and possess all the necessary machinery for antigen uptake, processing and presentation (58). Therefore, we also determined how EVs affected the levels of expression of surface MHC II on IgM^+^IgD^+^ B cells. For this, in some experiments, anti-MHC II β-chain ([mAb mouse IgG1 coupled to fluorescein isothiocyanate (FITC), 2 μg/ml] (59) was added in combination with the anti-IgM and anti-IgD mAbs. All the staining process was then performed as described above.

### 2.7 Evaluation of the effect of *B. subtilis* EVs on the antigen processing capacity of splenic IgM^+^ B cells

The antigen-processing capacity of IgM^+^ B cells was determined using the EnzChek protease assay kit (Invitrogen). For that, splenic leukocytes incubated with different doses of EVs or left unstimulated for 72 h as described above, were incubated with green fluorescent BODIPY DQ-CASEIN at 5 μg/ml for 1 h. DQ-CASEIN is a self-quenched form of fluorescently labelled casein (60), commonly used to study protease-mediated antigen processing because it exhibits bright green fluorescence upon proteolytic processing due to the released dye molecules (61). Negative controls in which cells were exposed to the same volume of the control preparation were also included (LB subjected to the same process of EV isolation). Afterwards, the cells were washed with staining buffer three times and labelled with the anti-trout IgM (clone 1.14) mAb coupled to APC (1 μg/ml) for 30 min at 4°C, rewashed and analyzed by flow cytometry as described above.

### 2.8 Proliferation of IgM^+^ B cells

The Click-IT^®^ EdU Alexa Fluor^®^ 488 flow cytometry assay kit (Life Technologies) was used to determine whether *B. subtilis* EVs could induce the proliferation of IgM^+^ B cells, following the manufacturer’s instructions. For this, splenic leukocytes were incubated with different doses of EVs or left unstimulated in the same conditions in 96-well plates for 72 h at 19°C. 24 h prior to analysis, 1 μM EdU (5-ethynyl-2′ -deoxyuridine) was added to the cultures and incubated until next day. Negative controls in which cells were exposed to the same volume of the control preparation were also included (LB subjected to the same process of EV isolation). The day after, the cells were collected, and their viability determined before fixation and permeabilization using the LIVE/DEAD^™^ Fixable Near-IR Dead Cell Stain kit (Invitrogen) for 30 min, following the manufacturer´s specifications. Cells were then washed and stained with the anti-trout IgM (1.14) mAb coupled to R-PE (1 μg/ml) for 30 min at 4°C. Cells were then fixed, permeabilized, and incubated with specific reagents to detect the incorporation of EdU into the DNA of proliferating cells following the manufacturer’s instructions. Samples were then analyzed on the flow cytometer as described above.

### 2.9 Enzyme-linked immunospot assay

ELISpot was used to evaluate the effect of the *B. subtilis* EVs on the total number of IgM-secreting B cells in splenic leukocyte cultures. For this, ELISpot plates containing Inmobilon-P membranes (Millipore) were activated with 70% ethanol for 30 s, coated with an anti-trout IgM mAb at 2 μg/ml in phosphate buffer saline (PBS) and incubated overnight at 4°C in agitation. To block non-specific binding to the membrane, plates were then incubated with 2% bovine serum albumin (BSA) in PBS for 2 h at room temperature (RT). Splenic leukocyte suspensions of individual fish that had been stimulated with different doses of *B. subtilis* EVs for 72 h at 19°C, with 1.5 x 10^6^ particles/ml of EVs from either *B. megaterium* or *B. subtilis* or left unstimulated in the same conditions, were then added to the wells in duplicate at a concentration of 1 x 10^5^ cells per well. Negative controls in which cells were exposed to the same volume of the control preparation were also included (LB subjected to the same process of EV isolation). After 24 h of incubation at 19°C, cells were washed away five times with PBS and plates were blocked again with 2% BSA in PBS for 1 h at RT. After blocking, biotinylated anti-IgM mAb at 1 μg/ml was added to the each well and incubated for 1 h at RT in agitation. Following additional washing steps (five times in PBS), the plates were developed using streptavidin-HRP (Thermo Scientific) at RT for 1 h, washed again with PBS and incubated with 3-amino-9-ethylcarbazole (Sigma Aldrich) for 30 min at RT in the dark. The substrate reaction was stopped by washing the plates with water. Once the membranes had dried, they were digitally scanned and the number of spots in each well determined using an AID iSpot Reader System (Autoimmun Diagnostika GMBH).

### 2.10 Statistical analysis

Data handling, statistical analyses and graphic representation were carried out using GraphPad Prism version 8 (GraphPad Software). Statistical analyses were performed using a two-tailed paired Student’s t-test. The differences between the mean values were considered significant on different degrees, where ∗ means *p* ≤ 0.05, ∗∗ means *p* ≤ 0.01, and ∗∗∗ means *p* ≤ 0.001. Furthermore, significant differences between mean values were denoted by letters in some experiments, with lowercase letters indicating differences among groups (*p* < 0.05).

## 3 Results

### 3.1 Physical characterization of *B. subtilis* EVs

**Figure 1:**
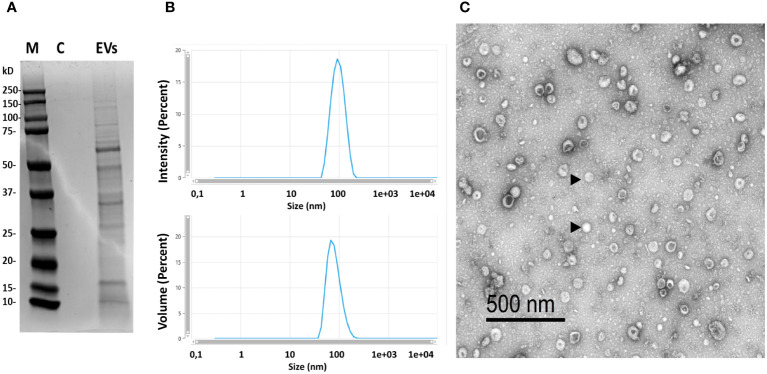
Characterization of the isolated EVs. **(A)** Protein profile of isolated EVs (M, marker; C, control consisting in the same volume of LB medium without bacteria that was subjected to purification process; EVs=purified EVs by ultracentrifugation at 100,000 x g for 1 h, 18 μg of protein were loaded). **(B)** Dynamic light scattering (DLS) profile of isolated EVs obtained in a Zetasizer Ultra device. **(C)** Transmission electron microscopy image of EVs of *B*. *subtilis* (representative EVs indicated with arrowheads).

We used different complementary approaches to confirm the purification of *B. subtilis* EVs and determine their integrity, size, shape, and concentration. When we observed the protein content by SDS-PAGE gel, a banding pattern was obtained that was not present in the control media subjected to the same purification process (**Figure 1A**). The size distribution of vesicles was confirmed by DLS measurements (**Figure 1B**), revealing a single peak size ranging from 90 to 100 nm in diameter, which provided an indication of the high purity of the EV preparation obtained. Moreover, EVs displayed the typical “round-shaped” morphology when visualized by TEM, with a diameter of up to 100 nm (**Figure 1C**). These structures were not visualized under the TEM or detected by DLS measurements when the culture media was subjected to the same purification protocol, confirming that they were produced by the bacteria. The typical yield obtained in the EV purification process was between 7x10^11^ to 1x10^12^ particles for each 500 ml of starting bacterial culture.

### 3.2 Transcriptional effects of the *B. subtilis* EVs on RTgutGC cells

We first studied how EVs affected the levels of transcription of a range of immune genes on the RTgutGC intestinal epithelial cell line. These included several genes coding for pro-inflammatory cytokines such as tumor necrosis factor α (*tnfa*), interleukin 1β (*il1b*) and *il8* (62). We also studied the transcriptional response of genes coding for antimicrobial peptides (AMPs), such as hepcidin and cathelicidin 2. Finally, we evaluated genes involved in intestinal barrier integrity and homeostasis, including genes involved in the intercellular tight junctions [e-cadherin (*cdh1*), claudin 3 (*claud3*), zonula occludens (*zo1*)] and a gene responsible for intestinal mucin production (*imuc*) (54, 55, 63, 64).

**Figure 2:**
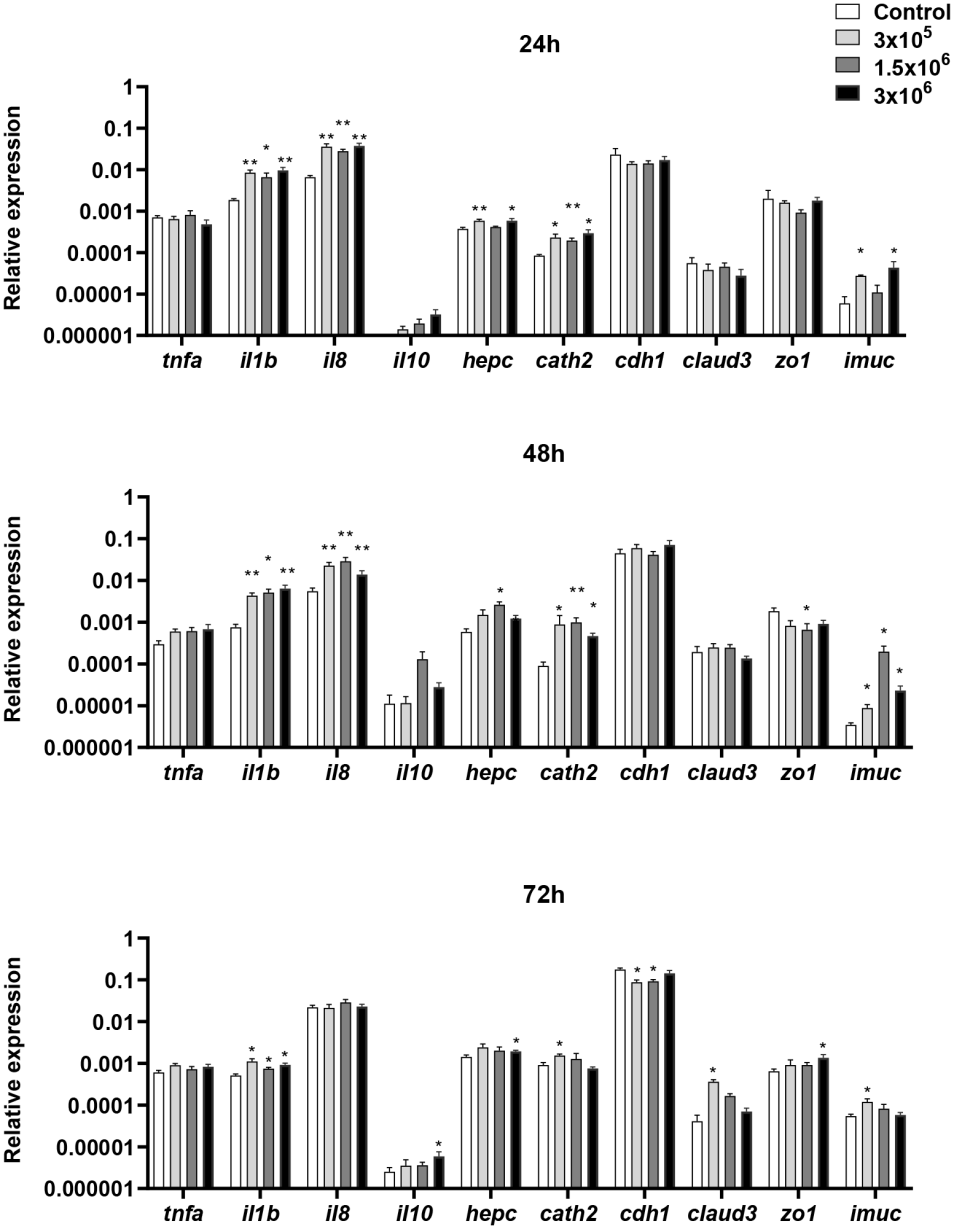
Transcriptional response of RTgutGC cells to different doses of *B*. *subtilis* EVs. RTgutGC cells were exposed to 3 x 10^5^, 1.5 x 10^6^ or 3 x 10^6^ per ml of *B*. *subtilis* EVs for 24, 48 and 72 h at 19°C. Thereafter, RNA was extracted and the levels of transcription of different genes analyzed by real-time PCR. Data are shown as the relative expression levels of the different genes normalized with the housekeeping gene *b-actin* (mean + SEM; n = 6). Asterisks denote transcription levels significantly different than those observed in cells exposed to *t*he same volume of culture media subjected to the same purification process *(*control) (*p ≤ 0.05 and **p ≤ 0.01).

The levels of transcription of *il1b* were significantly up-regulated in RTgutGC cells after 24, 48 and 72 h of stimulation with the different doses of EVs (**Figure 2**). Likewise, *il8* transcription augmented with the different doses of EVs, but only after 24 and 48 h of stimulation. On the other hand, the expression levels of *tnfa* remained stable throughout the experiment. Concerning the antimicrobial peptides, *B. subtilis* EVs up-regulated the levels of transcription of both hepcidin and cathelicidin 2 (**Figure 2**). In the case of cathelicidin 2, this up-regulation was observed in response to the three EV doses after 24 h and 48 h, whereas the up-regulation was only significantly achieved in response to specific EV doses for hepcidin. Lastly, the expression levels of *claud3* and *zo1* were up-regulated with some EV doses, mostly after 72 h of stimulation, whereas after 48 h of incubation, EVs provoked the down-regulation of *zo1* transcription (**Figure 2**). Similarly, the transcription of *cdh1* significantly decreased in response to the lower EV doses at the later time point (**Figure 2**). Finally, *imuc* was significantly up-regulated at all sampling times in response to some EV doses (**Figure 2**).

### 3.3 Effect of *B. subtilis* EVs on the transcriptomic response of splenic leukocytes

We then determined whether the *B. subtilis* EVs affected the transcriptional response of total splenic leukocyte cultures stimulated for 24 h with different concentrations of EVs. In this case, we studied the transcription of *tnfa*, *il1b* and *il8*, as well as *il10*, a gene mostly catalogued as anti-inflammatory (65) and the two AMPs tested before. Furthermore, in this case, we included CD4, CD8, FoxP3, GATA3 and Tbet as markers of different T cell populations, all forms of Ig genes and several genes known to be up-regulated during B cell differentiation, including *irf4* and the four isoforms of the *prdm1* genes coding for Blimp1 that are present in rainbow trout (66).

**Figure 3:**
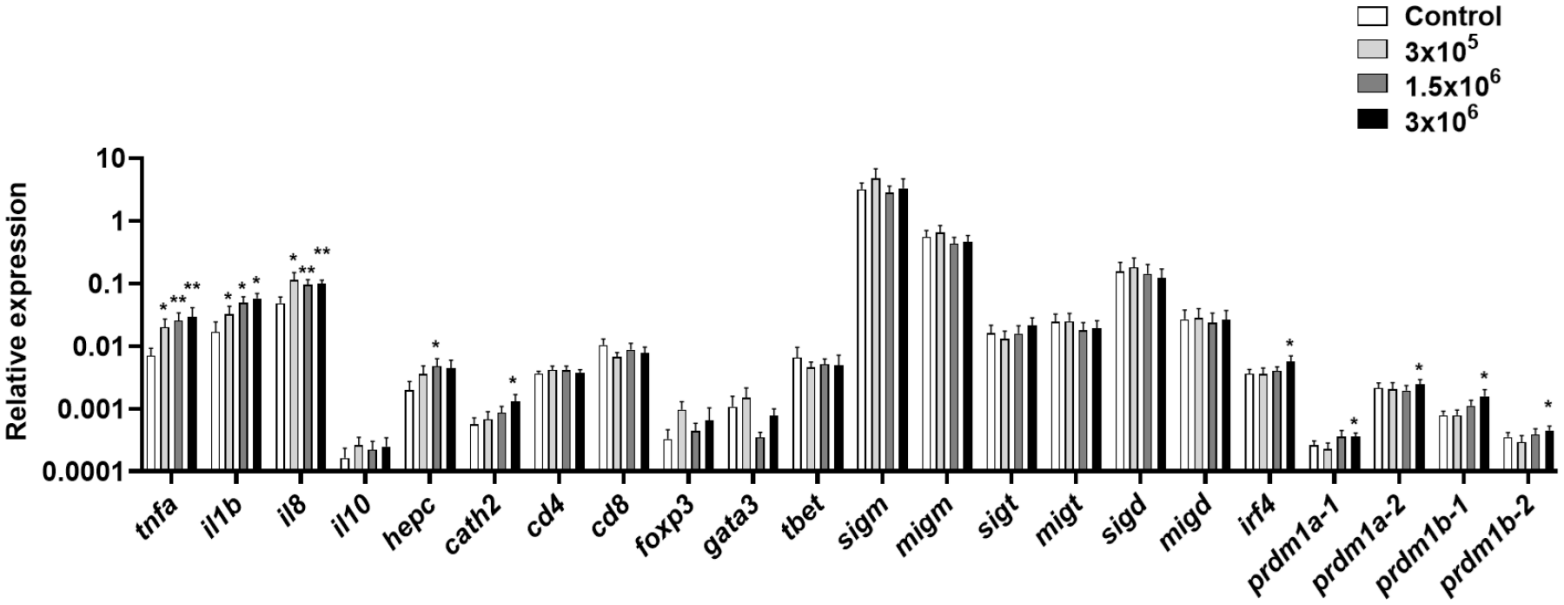
Transcriptional response of splenic leukocytes to different doses of *B*. *subtilis* EVs. Splenic leukocytes were exposed to 3 x 10^5^, 1.5 x 10^6^ or 3 x 10^6^ per ml of *B*. *subtilis* EVs and incubated for 24 h at 19°C. Thereafter, RNA was extracted and the levels of transcription of different genes analyzed by real-time PCR. Data are shown as relative expression levels of the different genes normalized with the housekeeping gene *b-actin* (mean + SEM; n = 6 independent fish). Asterisks denote significantly different transcription levels in treated groups compared to controls exposed to the same volume of culture media subjected to the same purification process (control) (*p ≤ 0.05 and **p ≤ 0.01).

We observed that mRNA levels of *tnfa*, *il1b* and *il8* were significantly up-regulated in splenic leukocytes exposed to any of the EV doses tested (**Figure 3**). Additionally, both hepcidin and cathelicidin 2 were also transcriptionally up-regulated in response to *B. subtilis* EVs at some doses (**Figure 3**). On the other hand, many genes related to B cell differentiation were transcriptionally increased in response to the highest EV dose, including *irf4*, *prdm1a-1*, *prdm1a-2, prdm1b-1*, and *prdm1b-2* (**Figure 3**). Genes related to T cell function were not significantly regulated in the presence of the EVs (**Figure 3**).

### 3.4 Effect of *B. subtilis* EVs on splenic B cell subsets

**Figure 4:**
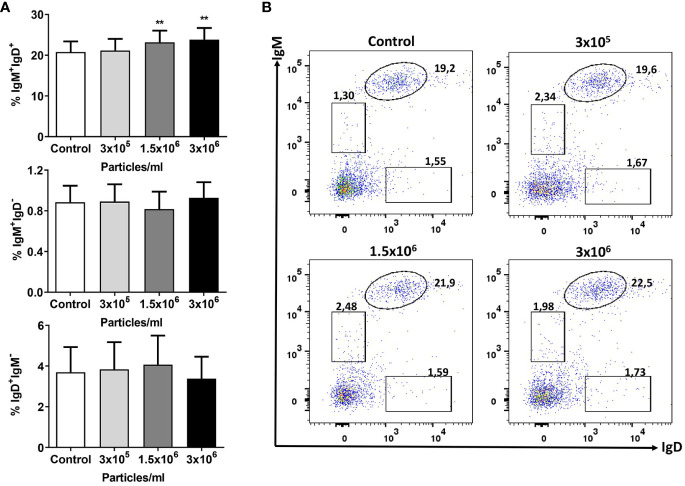
Flow cytometry analysis of B cell subsets after exposure to *B*. *subtilis* EVs. Splenic leukocytes stimulated or not for 72 h with the different EVs concentrations were labelled with specific mAbs anti-trout IgM and IgD and analysed by flow cytometry. **(A)** Graphs showing mean percentages of IgM^+^IgD^+^, IgM^+^IgD^-^ and IgD^+^IgM^-^ B cells among total lymphoid cells (mean + SEM; n =12 independent fish). **(B)** Representative dot plot from one fish in which the different B cell subsets are shown after incubation with the different EV concentrations. Asterisks denote significantly different values between control cells (exposed to the same volume of culture media subjected to the same purification process) and cells treated with EVs (**p ≤ 0.01).

Having seen an important transcriptional effect of *B. subtilis* EVs on genes related to B cell function in splenocyte cultures and given that *B. subtilis* had been shown to modulate the functionality of B cells in rainbow trout (49), we decided to investigate if *B. subtilis* EVs were capable of exerting similar effects. We first determined how the presence of EVs in the culture affected the percentages of IgM^+^IgD^+^, IgM^+^IgD^-^, and IgD^+^IgM^-^ B cell subsets. As previously reported, in rainbow trout spleen, IgM^+^IgD^+^ B cells make up for the vast majority of the IgM/D B cell population (52) and it was precisely this population, the one that significantly increased in the cultures in response to EV exposure (**Figure 4**). A small percentage of B cells in these cultures corresponded to IgM^+^IgD^-^ and IgD^+^IgM^-^ B cells, yet the survival or viability of these populations did not seem to be affected by *B. subtilis* EVs (**Figure 4**).

### 3.5 Effect of *B. subtilis* on MHC II surface expression and antigen processing capacities of splenic IgM^+^ B cells

**Figure 5:**
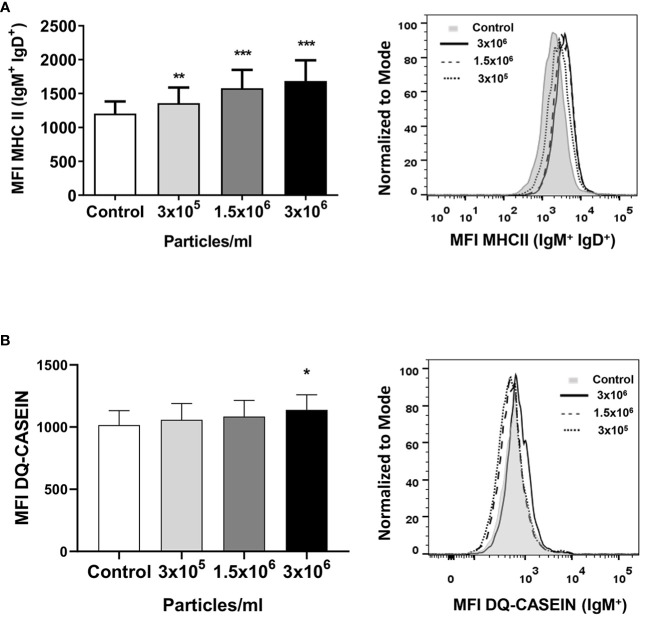
MHC II surface expression and antigen-processing capacity of splenic IgM^+^ cells after exposure to *B*. *subtilis* EVs. **(A)** Splenic leukocytes stimulated or not for 72 h with the different EV concentrations were labelled with mAbs anti-trout IgM, IgD and MHC II and analyzed by flow cytometry. A graph showing the mean fluorescence intensity (MFI) values of MHC II surface expression in IgM^+^IgD^+^ B cells (mean + SEM; n =12 independent fish) is shown along with a representative histogram. **(B)** In other experiments, these splenic leukocytes were incubated with DQ-casein (5 μg/mL) for 1 h at 19°C. Thereafter, cells were labelled with a mAb anti-trout IgM and analysed by flow cytometry. A representative histogram is shown along with a graph displaying the DQ-casein mean fluorescence intensity (MFI) values among IgM^+^ B cells (mean + SEM; n = 6 independent fish). Asterisks denote significantly different values between in cells treated with EVs when compared to control cells (exposed to the same volume of culture media subjected to the same purification process) (*p ≤ 0.05, **p ≤ 0.01 and ***p ≤ 0.001).

We also investigated the effects of exposure to *B. subtilis* EVs on the antigen presenting capacities of B cells. For this, we first studied the levels of MHC II on the surface of rainbow trout splenic cells. Our results clearly show that EVs significantly increased the levels of surface MHC II on IgM^+^IgD^+^ B cells from the spleen at all tested concentrations (**Figure 5A**). Additionally, the antigen-processing capacity of splenic IgM^+^ B cells was also significantly higher in cultures exposed to the highest concentration of EVs (**Figure 5B**).

### 3.6 Quantification of IgM-secreting cells by ELISpot

**Figure 6:**
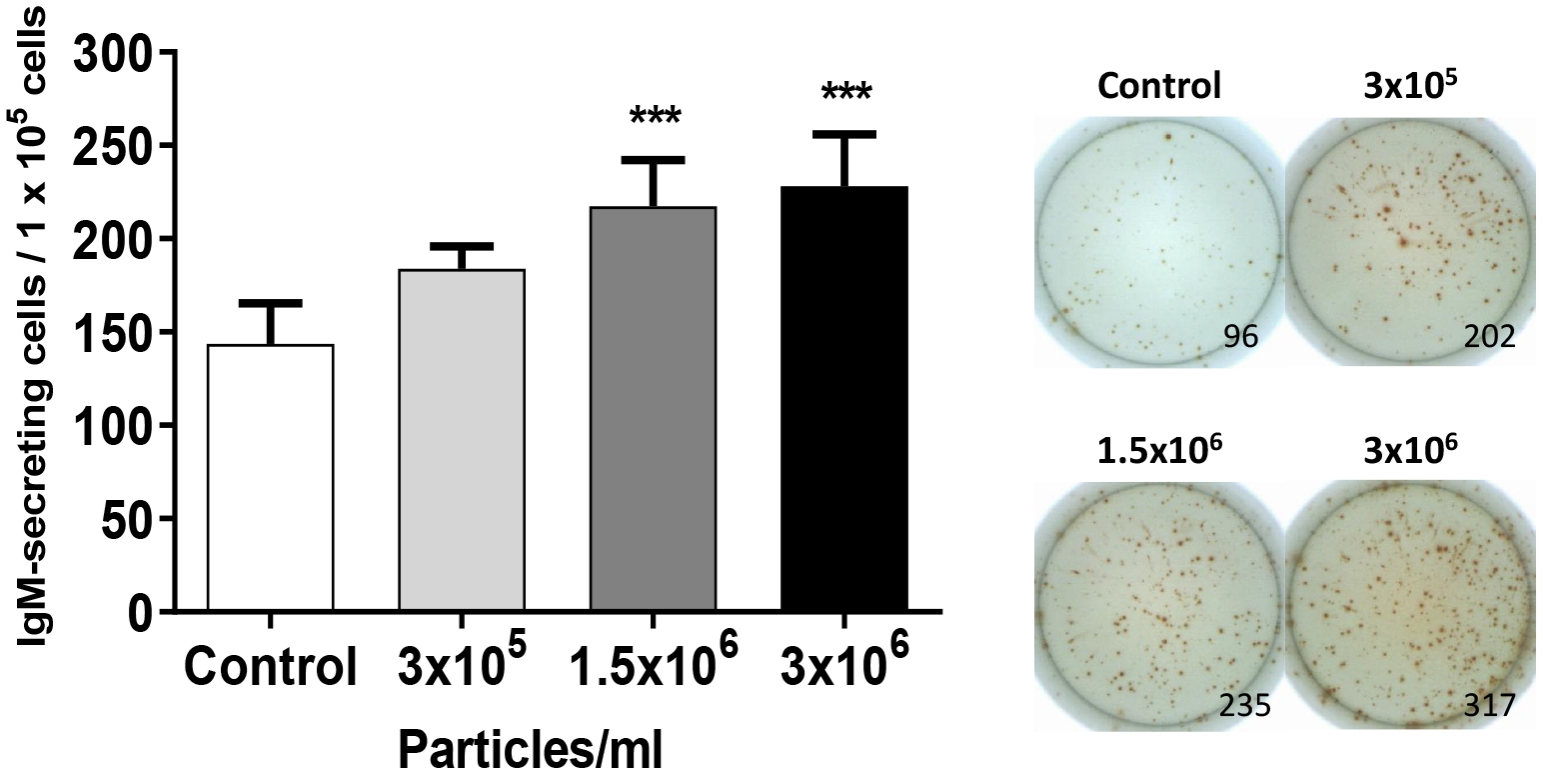
Quantification of IgM-secreting cells in splenocyte cultures after exposure to *B*. *subtilis* EVs. Splenic leukocytes (1x10^5^ cells) stimulated or not for 72 h with *B*. *subtilis* EVs were transferred to ELISpot plates pre-coated with a mAb anti-trout IgM for 24 h. After incubation, cells were washed and a biotinylated mAb anti-trout IgM was used to detect the number of spot-forming cells. Graph indicating mean number of spot-forming cells among 1 x 10^5^ cells (mean + SEM; n=12 independent fish) together with wells from a representative individual. Asterisks denote significantly different values between in cells treated with EVs when compared to control cells (exposed to the same volume of culture media subjected to the same purification process) (***p ≤ 0.001).

The fact that *B. subtilis* EVs up-regulated many genes related to B cell differentiation in splenic cultures suggested an effect on the number of IgM-secreting cells. To test this hypothesis, we evaluated the effects of EVs on the number of IgM-secreting in splenic leukocyte cultures by ELISpot. The number of IgM-secreting cells significantly augmented in splenic leukocyte cultures incubated with 1.5x10^6^ and 3x10^6^ EVs per ml when compared to the number of IgM-secreting cells found in non-stimulated cultures (**Figure 6**). This increase was not significant in splenic leukocyte cultures stimulated with the lowest dose of EVs.

### 3.7 The proliferative effect of *B. subtilis* EVs on spleen IgM^+^ and IgM ^-^ B cells

**Figure 7:**
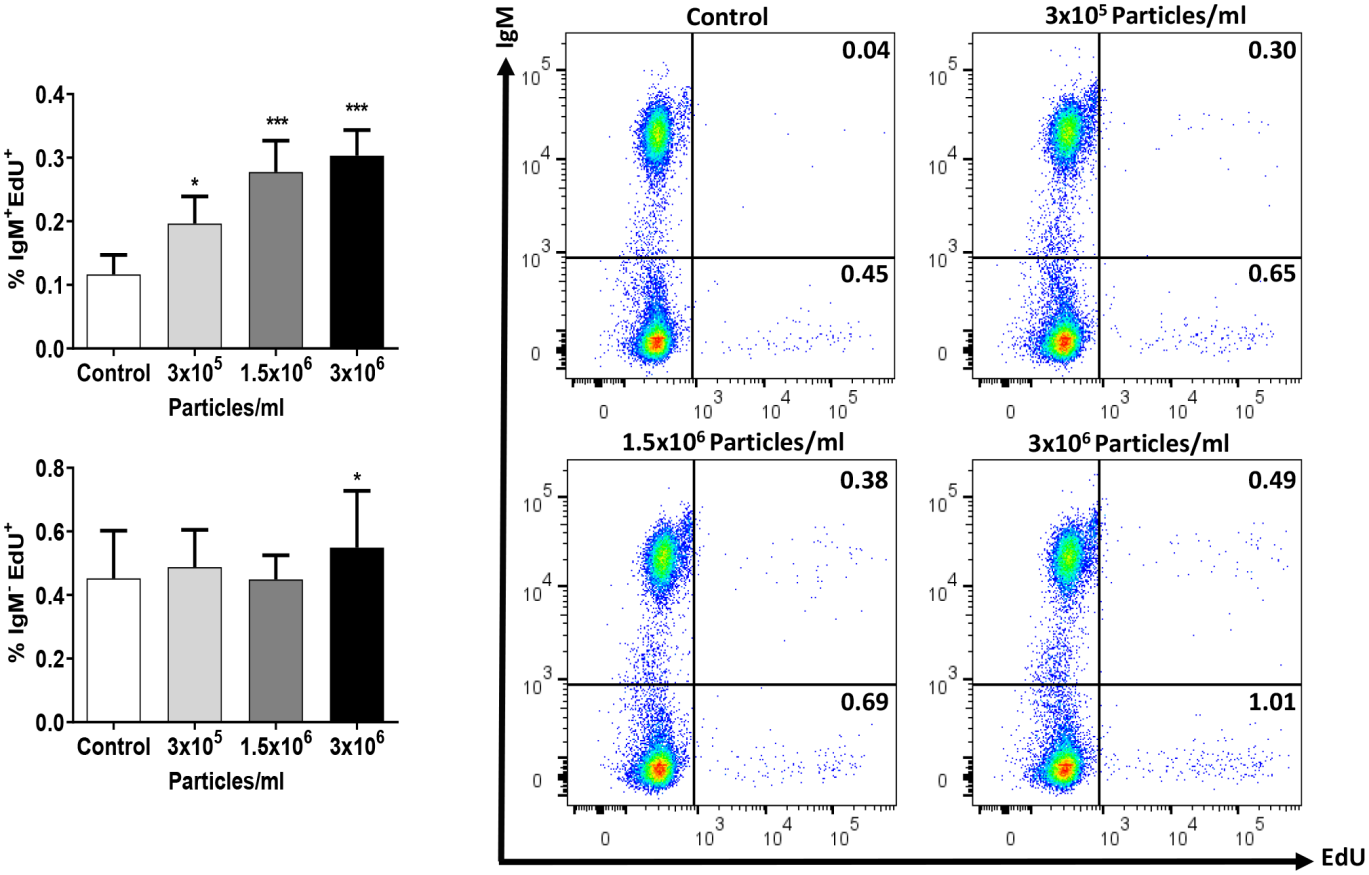
Proliferative effect of EVs on splenic IgM^+^ B cells. Splenic leukocytes were stimulated or not for 72 h with the different *B*. *subtilis* EV concentrations. After this time, the proliferation of IgM^+^ B cells and IgM^-^ cells was determined using Click-IT™ EdU cell proliferation kit Alexa Fluor™. The percentage of proliferating cells (EdU^+^) among IgM^+^ B cells and IgM^-^ cells is shown in graphs (mean + SEM; n = 12 independent fish) along with representative dot plots obtained in one individual. Asterisks denote significantly different values in treated groups compared to controls (exposed to the same volume of culture media subjected to the same purification process) (*p ≤ 0.05 and ***p ≤ 0.001).

To establish the lymphoproliferative potential of EVs derived from *B. subtilis*, we next studied the effects of EVs on the proliferation of IgM^+^ B cells in splenocyte cultures. Our results demonstrated that EVs were capable of provoking a slight but significant proliferation of IgM^+^ B cells in a dose-dependent fashion (**Figure 7**). Interestingly, the highest EV dose also provoked a significant increase in the number of proliferating IgM^-^ cells in these cultures (**Figure 7**).

### 3.8 Comparative effects of EVs from two *Bacillus* species

**Figure 8:**
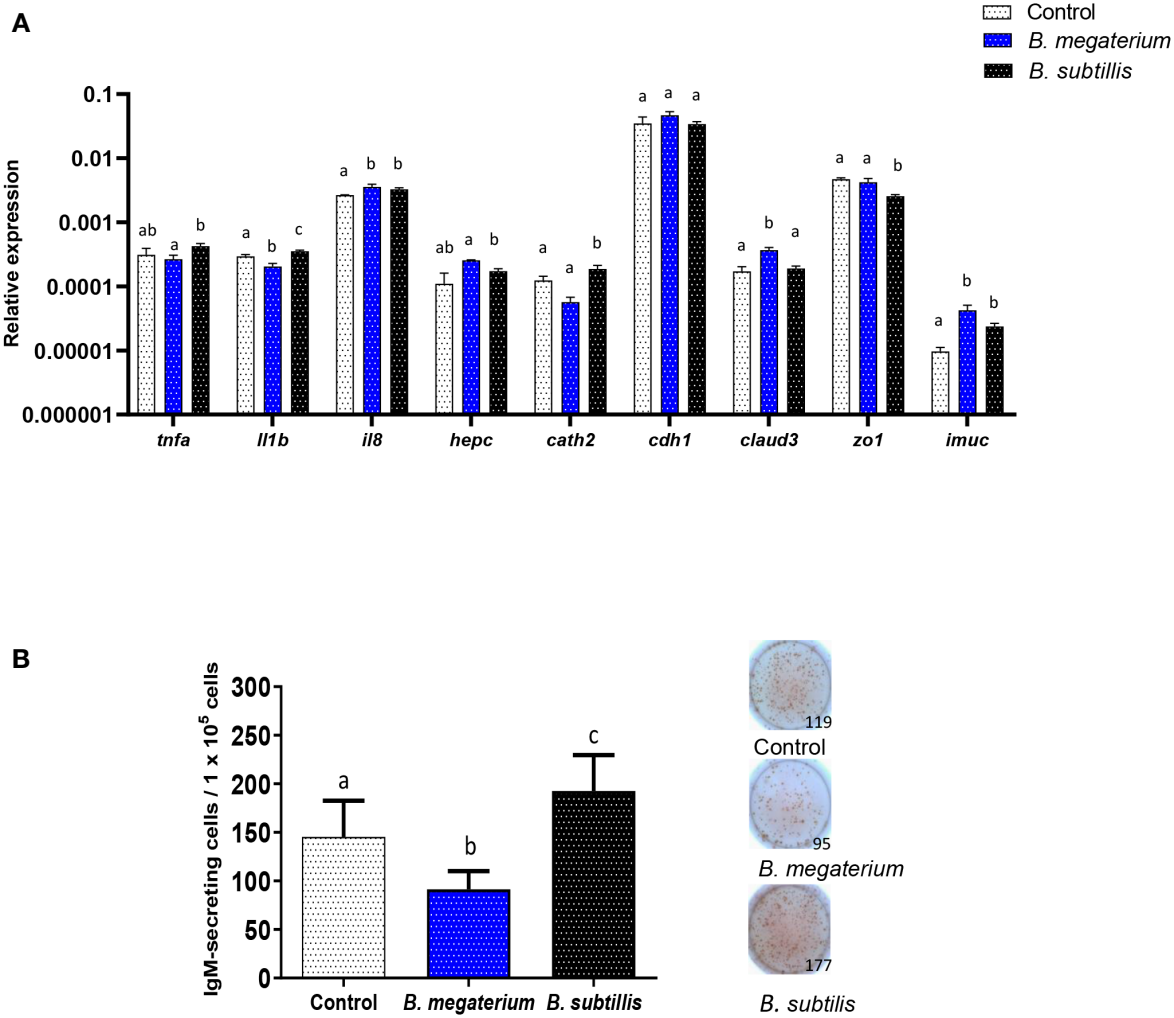
Comparative effects of EVs obtained from two *Bacillus* species. **(A)** Comparative transcriptional effects of *B*. *subtilis* or *B*. *megaterium* EVs on RTgutGC cells. For this, cells were incubated with 1.5 x 10^6^ particles/ml or with the same volume of control media subjected to the same purification process for 48 h at 19°C. Thereafter, RNA was extracted and the levels of transcription of different genes were analyzed by real-time PCR. Data are shown as relative expression levels of the different genes normalized with the housekeeping gene *b-actin* (mean + SEM; n = 5 independent fish). Different lowercase letters indicate significant differences among groups (*p* ≤ 0.05). **(B)** Quantification of IgM-secreting cells by ELISpot in splenocyte cultures exposed to *B*. *megaterium*, *B*. *subtilis* or to control media as described for **(A)** Cells were transferred to ELISpot plates pre-coated with mAb anti-trout IgM for 24 h. After incubation and washing cells, a biotinylated mAb anti-trout IgM was used to detect the number of spot-forming cells. A graph showing the quantification of spot-forming cells (mean + SEM; n = 9 independent fish) is included along with representative wells from one individual. Different lowercase letters indicate significant differences among groups (*p* ≤ 0.05).

To establish if the immunomodulatory effects provoked by *B. subtilis* EVs are specific to EVs from this probiotic species, we performed a series of experiments in which their effects were compared to those of EVs isolated from *B. megaterium*, another probiotic bacterium (67). We first evaluated their transcriptional effects on RTgutGC cells after 48 h, using the intermediate EV dose (1.5 x 10^6^ particles/ml) and studying the same genes studied before. The response to the two EVs was different for many genes. As seen before, *B. subtilis* EVs significantly increased the transcription of *il1b and cathelicidin 2*, while those of *B. megaterium* EVs significantly decreased them (*il1b*) or did not significantly affect them (*cathelicidin* 2) (**Figure 8A**). On the other hand, the transcription of *zo1* gene was negatively regulated by *B. subtilis* EVs, as described above, but not by *B. megaterium* EVs (**Figure 8A**). Moreover, *claud3* mRNA levels were significantly increased in response to *B. megaterium* EVs but not in response to *B. subtilis* EVs (**Figure 8A**). On the other hand, both types of EVs were able to stimulate significantly the transcription of cytokine *il8* and *imuc* (**Figure 8A**).

Finally, we compared the effects that a single dose of both types of EVs had on the number of IgM-secreting cells in splenic leukocyte cultures. As seen in the previous experiments (**Figure 6**), *B. subtilis* EVs provoked a significant up-regulation of the number of IgM-secreting cells when compared to non-stimulated cells (**Figure 8B**). However, this effect was not seen in response to *B. megaterium* EVs which even provoked a significant decrease in the number of IgM-secreting cells in the cultures (**Figure 8B**).

## 4 Discussion

In the past recent years, a growing interest in the development of novel immunostimulants and vaccines that may be administered orally to aquacultured fish has arisen for practical reasons (68). Bacterial EVs, specially OMVs have emerged as a promising mucosal vaccination platforms and adjuvants in mammals (69), where they have been proven capable of conferring protection against pathogens by activating both adaptive and cellular immune responses (19, 30, 31, 37, 39, 70–72). Yet, only a scarce number of studies have investigated the effectiveness of EVs derived from Gram-positive bacteria as adjuvants or vaccination platforms (28, 31, 73).

In fish, although a few studies have investigated the production of EVs by some fish-related bacteria and their effects on cells, very few works have provided evidence of their potential as immunostimulants or vaccine adjuvants. In this context, we decided to investigate whether EVs from the probiotic *B. subtilis* have similar immunomodulatory properties to those seen in response to the bacterial strain. *B. subtilis* has been found to colonize the gastrointestinal tract (74), and reported transcytosis of *B. subtilis* EVs by a cell model, which imitates gastrointestinal epithelium, suggested that EVs from *B. subtilis* can reach the bloodstream and thereby extra-intestinal tissues and organs (75). In addition, oral administration of *B. subtilis* has been shown to regulate the host immune system by enhancing both humoral and innate parameters (76). Consequently, the continued administration of *B. subtilis* was able to enhance disease resistance in species such as rainbow trout (77), white shrimp (*Litopenaeus vannamei*) or Dabry’s sturgeon (*Acipenser dabryanus*) (78, 79). Moreover, even a single administration of *B. subtilis* was shown to induce the transcription of genes involved in inflammation, antimicrobial genes, and genes involved in T cell responses in the rainbow trout intestine and systemic immune organs (49). Additionally, the capacity of *B. subtilis* to activate different functionalities of B cells has also been recently demonstrated in rainbow trout (49).

Although mammalian strains of *B. subtilis* were previously shown to produce EVs containing lipoproteins and proteins related to antibiotic resistance (80, 81), the immunomodulatory capacities of these EVs have never been established. The *B. subtilis* ABP1 strain used in the current work isolated from European seabass, released nanosized extracellular vesicles with diameters ranging from 90 to 100 nm. The diameter size detected by TEM and DLS was similar despite the possible over/underestimation that can sometimes occur with these techniques (82). Additionally, the sizes are similar to those previously found in EVs from mammalian *B. subtilis* strains and other *Bacillus* species (24, 31, 80). However, the DLS technique revealed only one population, while EVs of alternative sizes can be produced by other *Bacillus* strains (80). The banding pattern obtained in the SDS-PAGE gel was also similar to that previously reported (80).

Initially, we used the established RTgutGC cell line to test the transcriptional effects provoked by *B. subtilis* EVs, to then determine their effects on splenic leukocytes. The RTgutGC cell line has characteristic features of functional intestinal epithelial cells and has been widely used to predict immune effects on fish intestinal cells (49, 83, 84). Both experiments confirmed the immunostimulatory properties of *B. subtilis* EVs, with the dose-dependent up-regulation of *il1b* and *il8* transcription, and stimulatory effects on *tnfa* mRNA levels in the case of splenic leukocyte cultures. Similar effects on the transcription of pro-inflammatory genes have been reported for different OMVs (85, 86). Furthermore, in RTgutGC cells but not in isolated splenic leukocytes, EVs induced the transcription of *cathelicidin 2* and *hepcidin*, two AMPs with known antibacterial properties (87) Similarly, OMVs have been demonstrated to induce the production of certain antimicrobial compounds (88). Likewise, stimulation of RTgutGC cells with two strains of *B. subtilis* was shown to elicit the transcription of several AMP genes (49).

In RTgutGC cells, we also evaluated the effects of the EVs on the transcription of a range of genes related to intestinal barrier function, integrity and homeostasis. *B. subtilis* EVs modulated the transcription of *cdh1*, *claud3*, *zo1* and *imuc*, which suggests that these EVs might be able to modulate mucus production and permeability in the rainbow trout intestine. These results are in agreement with those reported by Alvarez and collaborators (51) in which proteins related to permeability and junction functions were transcriptionally increased after EV stimulation. Interestingly, EV released by microbiota species has also been shown to modulate the permeability of epithelial barriers, explaining their positive impact on health and disease resistance (89). This increased permeability would favor the transporting and delivering of effector molecules into host cells, thus modulating host signaling pathways and cellular processes (90).

In the case of splenic leukocyte cultures, we also found that *B. subtilis* EVs modulated the transcription of many genes related to B cell functionality, especially genes that drive the differentiation process from naïve B cells to antibody-secreting cells, first plasmablasts and eventually plasma cells. These included *irf4* and different homologues of mammalian *prdm1*, a gene that codes for Blimp 1 (66, 91). These results prompted us to study further the effects that *B. subtilis* EVs had on rainbow trout splenic B cells. We first observed that the EVs increased the percentage of IgM^+^IgD^+^ B cells in the cultures while having non-significant effects on other minor B cell subsets. This demonstrated that *B. subtilis* EVs are not toxic for B cells and suggest positive effects on their survival. Additionally, there was a modest but significant proliferation of IgM^+^ B cells in these cultures in response to the EVs.

As suggested by the transcriptional effects reported, *B. subtilis* EVs significantly increased the number of cells secreting IgM in the cell cultures in a dose-dependent fashion. These results altogether suggest that *B. subtilis* EVs on their own are capable of promoting the differentiation of B cells to plasmablasts/plasma cells. This differentiation of IgM^+^ B cells has been reported in rainbow trout in response to *B. subtilis* (49) and pathogenic bacterial species such as *Aeromonas salmonicida* (92).

When B cells differentiate into plasmablasts/plasma cells, they usually decrease their antigen-presenting capacities and consequently MHC II surface expression (93). Yet, recent reports in mammals have shown that in some cases, such as when induced by thymus-independent antigens, plasma cells can retain MHC II levels (94). In fish, recent evidence gathered by our group has shown that although in some cases the differentiation of IgM^+^ B cells implies a reduction of MHC II (95), there are many cases in which IgM^+^ differentiate to plasmablasts/plasma cells and simultaneously increase their antigen-processing capacities and MHC II expression (96, 97). Similarly, *B. subtilis* EVs seemed to simultaneously increase surface MHC II and antigen processing while significantly increasing IgM secretion.

Finally, we performed a series of studies to establish if the effects that we observed in response to *B. subtilis* EVs were specific for EVs of this bacterial species or could be extended to other species. For that, we isolated EVs in parallel from *B. megaterium*, a bacterium that has also shown probiotic properties (67, 98, 99) and for which immunomodulatory effects have been reported. Thus, for example, Nile tilapia (*O. niloticus*) fed with a probiotic mixture containing *B. megaterium* showed an enhanced transcription of *il1b* and *tnfa* (100). However, in our study, *B. megaterium* failed to up-regulate the transcription of both pro-inflammatory cytokines in RTgutGC cells, while *B. subtilis* EVs did in the same conditions. This increased immunomodulatory ability of *B. subtilis* EVs was even more pronounced in the case of the antimicrobial peptide gene *cathelicidin 2*. In contrast, *B. megaterium* EVs increased *claud3* transcription whereas *B. subtilis* EVs did not. Additionally, both EVs similarly regulated *imuc* transcription in RTgutGC cells. This aligns with previous findings (99), in which different intestinal parameters were modulated in fish fed with different concentrations of *B. megaterium*. Yet the most drastic difference between the two EVs was that observed when studying the number of IgM-secreting cells in splenocyte cultures, since *B. subtilis* EVs significantly increased the number of IgM-secreting cells whereas *B. megaterium* EVs decreased it. These results confirm that EVs derived from diverse *Bacillus* species exhibit quite different abilities to modulate the immune response.

In conclusion, our findings suggest that EVs derived from *B. subtilis* are capable of stimulating and modulating functions related to both innate and adaptive immune functions in rainbow trout cells. This capacity of EVs to enhance the immune system points to their great potential as immunostimulants, adjuvants or even vaccination vehicles for use in aquaculture.

## Data availability statement

The raw data supporting the conclusions of this article will be made available by the authors, without undue reservation.

## Ethics statement

The animal study was approved by Consejo Superior Investigaciones Científicas (CSIC) Ethics Committee. The study was conducted in accordance with the local legislation and institutional requirements.

## Author contributions

SV-G: Formal analysis, Investigation, Methodology, Writing – review & editing. NN-O: Formal analysis, Investigation, Methodology, Writing – original draft. EM: Formal analysis, Investigation, Methodology, Writing – review & editing. CS: Methodology, Resources, Writing – review & editing. FD: Investigation, Methodology, Writing – review & editing. PD-R: Formal analysis, Investigation, Methodology, Writing – review & editing. CT: Conceptualization, Formal analysis, Funding acquisition, Validation, Writing – original draft.
