# Supplementary material for: Immunomodulatory properties of Bacillus subtilis extracellular vesicles on rainbow trout intestinal cells and splenic leukocytes
Source: Front Immunol. 2024 May 7;15:1394501. doi: 10.3389/fimmu.2024.1394501 (PMC11106384; doi:10.3389/fimmu.2024.1394501)
Supplement: Supplementary file 1 [file DataSheet_1.pdf]

Gene	Forward primer sequences	Reverse primer sequences
b-actin	TCCTTCCTCGGTATGGAGTCT	TTACGGATGTCCACGTCACAC
tnfa	CCACACACTGGGCTCTTCTT	GTCCGAATAGCGCCAAATAA
il1b	GACATGGTGCGTTTCCTTTT	ACCGGTTTGGTGTAGTCCTG
il8	ATTGAGACGGAAAGCAGACG	CTTGCTCAGAGTGGCAATGA
il10	GCTATGGACAGCATCCTGAAGTT	CTGCATTGGACGATCTCTTTCTT
hepc	GCTGTTCCTTTCTCCGAGGTGC	GTGACAGCAGTTGCAGCACCA
cath2	ACATGGAGGCAGAAGTTCAGAAGA	GAGCCAAACCCAGGACGAGA
cdh1	ACTATGACGAGGAGGGAGGT	TGGAGCGATGTCATTACGGA
claud3	AGGCAACGACGCTACATCAA	GAAACCCAAGCAATGCGTCA
zo1	GCTGTTCCTCCTAGACCTT	TCACCCACATCTGACTCTAC
imuc	TCAACACATTCTCTGACACC	GGCAGTTACTGTACCAAGTC
cd4	CCTGCTCATCCACAGCCTAT	CTTCTCCTGGCTGTCTGACC
cd8	AGTCGTGCAAAGTGGGAAAG	GGTTGCAATGGCATACAGTG
foxp3	CCAGAACCGAGGTGGAGTGT	TGACGGACAGCGTTCTTCCA
gata3	CCCATCGGTGCTAAACGAACA	GCTGTGGTGCTGCATTGCTT
tbet	GTTCTGCAGTCGCTTCATAAGTACC	CTATGAATTGGGTCTCTGGGAAGAC
sigm	TACAAGAGGGAGACCGGAGGAGT	CTTCCTGATTGAATCTGGCTAGTGGT
migm	CCTACAAGAGGGAGACCGATTGTC	GTCTTCATTTCACCTTGATGGCAGT
sigt	CATCAGCTTCACCAAAGGAAGTGA	TCACTTGTCTTCACATGAGTTACCCGT
migt	TCGAAGTCCACGGCGAACA	GTGTTCTTCACCGCTTCATCTTGAA
sigd	TGGCACGCCAGGATTTGAC	TCAGAATTGAGTGAACGGACAGACA
migd	CAGGAGGAAAGTTCGGCATCA	CCTCAAGGAGCTCTGGTTTGGA
irf4	CATGGCCACCCTTTCTGACTTCC	GCATACCCCTGCAGCTCAGTGA
prdm1a-1	GGGGGTAGAGGGCACAGC	CAGCGCCCCAGTCAAGATA
prdm1a-2	CATTCGGCCCTATGTGTGG	CCCCTCGGTAGTCAACATGG
prdm1b-1	ACGACGTCATCGCACACTTC	CCCTCCCCAAACGGGTAA
prdm1b-2	GGCTAGCGTGGTACGCTTCT	ACAAGGGTCCGTCCACTTTG

Table S1: List of primers used in this study for RT-PCR gene expression analysis.

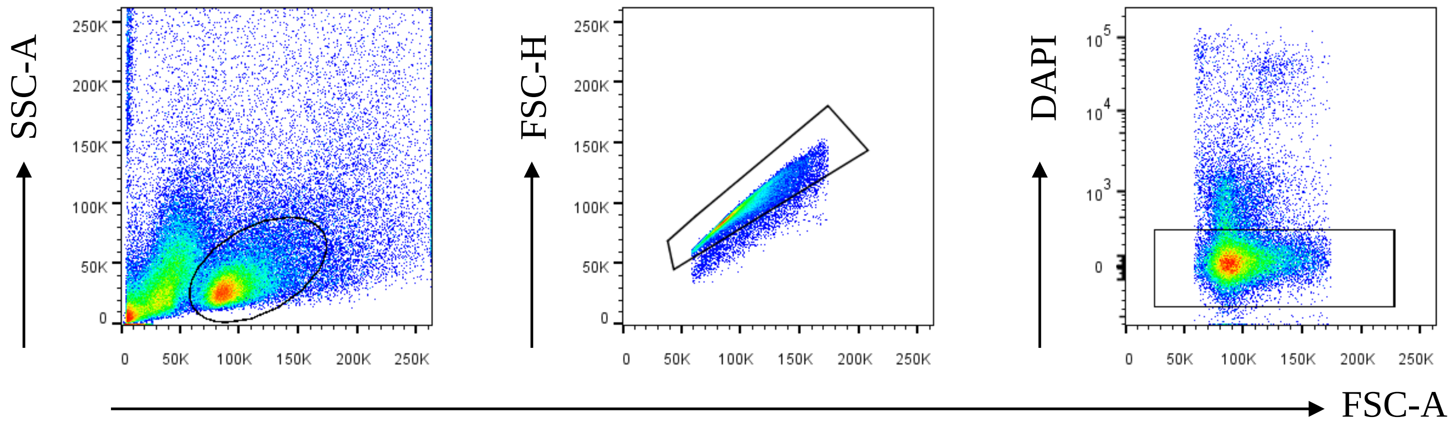

Figure S1: Gating strategy (Related to Figures 4,5,7). Splenic leukocytes were isolated from the trout spleen. FSC/SSC profiles including a defined gate for lymphoid cells are shown. FSC-H/FSC-A profiles within the lymphoid gate indicate singlets. DAPI-negative cells within the singlet gate were gated to select live cells.
